# Endothelial Regulator of Calcineurin 1 Promotes Barrier Integrity and Modulates Histamine-Induced Barrier Dysfunction in Anaphylaxis

**DOI:** 10.3389/fimmu.2017.01323

**Published:** 2017-10-20

**Authors:** Constanza Ballesteros-Martinez, Nerea Mendez-Barbero, Alma Montalvo-Yuste, Bettina M. Jensen, Aída Gomez-Cardenosa, Lotte Klitfod, María Garrido-Arandia, Gloria Alvarez-Llamas, Carlos Pastor-Vargas, Fernando Vivanco, Lene Heise Garvey, Javier Cuesta-Herranz, Lars K. Poulsen, Vanesa Esteban

**Affiliations:** ^1^Department of Immunology, IIS-Fundación Jiménez Díaz, Autonomous University of Madrid, Madrid, Spain; ^2^Department of Vascular Physiopathology, IIS-Fundación Jiménez Díaz, Autonomous University of Madrid, Madrid, Spain; ^3^Allergy Clinic, Gentofte Hospital, Copenhagen University Hospital, Hellerup, Denmark; ^4^Department of Allergy, Fundación Jiménez Díaz, Madrid, Spain; ^5^Surgery Department, Gentofte Hospital, Copenhagen University Hospital, Hellerup, Denmark; ^6^Center for Plant Biotechnology and Genomics, Technical University of Madrid, Madrid, Spain

**Keywords:** anaphylaxis, endothelial cells, vascular permeability, regulator of calcineurin 1, histamine

## Abstract

Anaphylaxis, the most serious and life-threatening allergic reaction, produces the release of inflammatory mediators by mast cells and basophils. Regulator of calcineurin 1 (Rcan1) is a negative regulator of mast-cell degranulation. The action of mediators leads to vasodilation and an increase in vascular permeability, causing great loss of intravascular volume in a short time. Nevertheless, the molecular basis remains unexplored on the vascular level. We investigated Rcan1 expression induced by histamine, platelet-activating factor (PAF), and epinephrine in primary human vein (HV)-/artery (HA)-derived endothelial cells (ECs) and human dermal microvascular ECs (HMVEC-D). Vascular permeability was analyzed *in vitro* in human ECs with forced Rcan1 expression using Transwell migration assays and *in vivo* using Rcan1 knockout mice. Histamine, but neither PAF nor epinephrine, induced Rcan1-4 mRNA and protein expression in primary HV-ECs, HA-ECs, and HMVEC-D through histamine receptor 1 (H1R). These effects were prevented by pharmacological inhibition of calcineurin with cyclosporine A. Moreover, intravenous histamine administration increased Rcan1 expression in lung tissues of mice undergoing experimental anaphylaxis. Functional *in vitro* assays showed that overexpression of Rcan1 promotes barrier integrity, suggesting a role played by this molecule in vascular permeability. Consistent with these findings, *in vivo* models of subcutaneous and intravenous histamine-mediated fluid extravasation showed increased response in skin, aorta, and lungs of *Rcan1*-deficient mice compared with wild-type animals. These findings reveal that endothelial Rcan1 is synthesized in response to histamine through a calcineurin-sensitive pathway and may reduce barrier breakdown, thus contributing to the strengthening of the endothelium and resistance to anaphylaxis. These new insights underscore its potential role as a regulator of sensitivity to anaphylaxis in humans.

## Introduction

Anaphylaxis is a potentially lethal, rapid-onset allergic reaction, and is known to be the most aggressive manifestation of allergic disorders (1). Anaphylactic reactions trigger a broad range of symptoms affecting different organs and bodily systems. The severe alterations described in human anaphylaxis involve the cardiovascular system and include loss of peripheral vascular resistance (vasodilation) and exacerbated vascular extravasation. These physiological processes are associated with low blood pressure, reduced venous return, and decreased cardiac output. Furthermore, bronchoconstriction and pulmonary/coronary-artery vasoconstriction commonly occur in the thoracic cavity, which contributes to widespread circulatory collapse (2–4). The clinical features of anaphylaxis have been well-described and classified, though investigations into its molecular signaling dynamics remain scarce in humans. To date, this hypersensitivity event has been considered a disorder of the immune system. However, immunological mechanisms do not fully explain the versatility of the events that take place during a reaction (5). In order to obtain a better understanding of anaphylaxis, we searched for new strategies, including the study of vascular mechanisms.

Molecules released mainly by active mast cells and basophiles interact with the vascular endothelium and the smooth muscle layers, destabilizing the endothelial barrier and modifying essential vascular contractile functions (tone) in vessels and airways (6). Tryptase, histamine, and platelet-activating factor (PAF) are only two such relevant biochemical mediators found in the serum of anaphylaxis patients (7). In cells, histamine binds to four types of receptors coupled to G proteins (H1-4R), and these receptors are widely distributed across tissues and leads to signaling through numerous molecular pathways. Type-1 and type-2 histamine receptors (H1R and H2R) have been widely investigated due to the fact that they mediate the intracellular signaling associated with second messengers such as Ca^2+^ and cAMP, which regulate vascular permeability, vasodilation, and bronchoconstriction (8, 9). In order to control these homeostatic disorders, the first-line treatment for anaphylaxis is intramuscular injection of epinephrine, which acts *via* α- and β-adrenergic receptors, triggering intracellular mechanisms in cardiac and smooth vascular cells (10). Their potent vasoconstrictor actions combined with fluid therapy are effective when administered promptly (11). Moreover, the second messenger cAMP is mediated by activation of β-adrenergic receptor signaling and contributes to the maintenance of endothelial barrier properties under baseline conditions (12).

There is growing interest in understanding the vascular permeability and vasodilation that occur during anaphylaxis. Different endothelial molecular pathways have been described as key targets for anaphylaxis due to their implication in the disruption of endothelial integrity or vascular tone modulation (13, 14). In humans, changes in vascular permeability during anaphylaxis may lead to a transfer of 50% of the intravascular fluid into the interstitial space within 10 min (3). At the molecular and cellular level, it is well known that histamine induces rapid and transient processes, which disrupt the endothelial barrier, thereby allowing the leakage of fluids, mainly in venules (15). Furthermore, endothelial cells (ECs) participate in physiological processes that regulate not only the capillary component but also peripheral vascular resistance and homeostasis. This fact is a focus of research in vascular permeability modulation (16), and vascular wall components are also essential in regulating leakage and peripheral vascular resistance in anaphylaxis. Mechanistically, a cellular counterbalance between contractile and adhesive forces must exist to maintain the stability between cells and prevent the rupture of the endothelial barrier (17). It has been reported that ECs contribute to the widespread effects observed in anaphylaxis through synthesis and the release of substances, including nitric oxide (NO) and mediators generated from the arachidonic acid cascades (18). However, mast cells are the main cellular source recognized to date, and are major releasers of prostaglandins and leukotrienes eliciting anaphylaxis reactions (19, 20).

Histamine binding to H1 receptors activates PLCβ and elevates intracellular Ca^2+^, both of which determine the signaling pathways which regulate inflammatory processes. *Via* Ca^2+^-dependent mechanisms, changes take place in cytoskeleton proteins or junction structures that determine cellular permeability and contractility (21). One of the most sensitive downstream effectors of Ca^2+^ is the ubiquitously expressed serine/threonine protein phosphatase calcineurin (22). Activation of calcineurin contributes to immune response signaling by members of the nuclear factor of activated T-cells (NFAT) family (23). Calcineurin activity can be inhibited by the immunosuppressant cyclosporine A (CsA) which forms a complex with cyclophilin A to bind and competitively inhibit calcineurin phosphatase activity (24). Endogenous regulation of calcineurin is mediated by members of the regulator of calcineurin (Rcan) family, and Rcan1 is the only such molecule regulated by Ca^2+^/calcineurin (25). The RCAN1 gene contains seven exons that can generate several transcripts resulting from differential promoter use and first exon choice. The two major transcriptional products for Rcan1 are isoforms, including exons 1 + 5–7 (Rcan1-1) and isoform 4 (Rcan1-4) with exons 4 + 5–7, which produce proteins with 252 and 197 amino acids, respectively (26, 27).

Divergent functions have been reported for both Rcan1-1 and Rcan1-4. While different inducers of Ca^2+^ selectively upregulate Rcan1-4, few stimuli have been described as modulators of Rcan1-1 expression. A role for apoptosis is attributed to Rcan1-1 in response to glucocorticoids, and relevant studies have linked Rcan1-1 to Huntington disease (28, 29). Rcan1-4 is upregulated by increases in Ca^2^ or in response to a variety of signals, including cytokines, hormones, hydrogen peroxide, and stress (30). Functionally, it has been widely described as an anti-inflammatory, anti-angiogenic agent and modulator of cardiovascular pathologies (31, 32). Due to its influence in regulating calcineurin activity, Rcan1 is involved in a broad range of cellular systems and biological processes. Extensive investigations have provided insights into EC signaling, describing Rcan1 as a potential therapeutic target in vascular inflammation (33). VEGF and thrombin have been reported to be the major inducers of Rcan1 in ECs, while angiotensin II induces Rcan1 expression in vascular smooth muscle cells (34–36).

Given the crucial role exerted by mediators on the vascular wall in anaphylaxis, we assessed the impact of anaphylaxis on Rcan1 expression in human ECs, as well as its functional involvement in vascular permeability and cell dilation. This study evaluates Rcan1 expression in human ECs in response to mediators of anaphylaxis and, more specifically, the involvement of histamine receptors involved in Rcan1 expression. Moreover, we studied the permeability effects of Rcan1 causing either endothelial barrier rupture or strengthening in response to mediators of anaphylaxis and also analyzed the plausible endothelial mechanisms exerting these functions.

## Materials and Methods

### *In Vitro* Human Cell Cultures

Human dermal microvascular ECs (HMVEC-D) were acquired from Lonza. Human vascular endothelial vein and artery cells were isolated from the macroscopically healthy part of intact saphenous veins harvested from patients undergoing bypass surgery/high ligation of varicose veins. The study was approved by the research ethics committees of the Gentofte and FJD hospitals, and written informed consent was obtained from all patients. Briefly, after removal of the connective tissue, the vein was opened longitudinally and the endothelium was isolated by digestion with 0.1% type I collagenase (Gibco) in PBS for 30 min at 37°C. Similarly, artery specimens were incubated with the digestion buffer O.N at 37°C. Reactions were stopped and cells were collected by centrifugation and grown in DMEMF12 media supplemented with 10 U/ml heparin, 30 g/ml ECGF, 100 U/ml penicillin, 100 g/ml streptomycin, and 15% (v/v) heat-inactivated fetal bovine serum. Cells were seeded in a plate previously coated with 0.5% sterile gelatin and maintained in a humidified atmosphere of 5% CO_2_ in air at 37°C. After 5–7 days of incubation, human vein or artery ECs (abbreviated herein as HV-EC/HA-ECs) were selected with human CD31 antibody. Next, the secondary antibody associated with magnetic beads (Dynabeads^®^ anti-mouse IgG from CELLection ™ Pan Mouse IgG Kit) was incubated for 30 min at 4°C with constant shaking. ECs were seeded on plates previously treated with 0.5% gelatin. Once in confluence, primary cell cultures were FBS 0.5% starved for 18 h before the experiments were performed. All experiments were performed during passages 3–7. A similar protocol was applied to the artery specimens.

### Reagents

Histamine, PAF, epinephrine, and the histamine receptor antagonists diphenhydramine hydrochloride (H1RB) and famotidine (H2RB) were obtained from Sigma.

### Protein Extraction and Immunoblot Analysis

Endothelial cells were lysed with buffer containing Tris, NaCl, EDTA, EGTA, Triton X-100, NP40, protease inhibitors, PMSF, and DTT. Cellular lysates were shaken for 15 min and centrifuged at 12,000 rpm at 4°C. After stimulation with reagents or sera, ECs were washed with ice-cold PBS and protein extracts obtained as previously described (36). Rabbit polyclonal anti-Rcan1 primary antibody was used at a ratio of 1/1,000 (Sigma). Analysis of images was carried out using the ImageJ program. Protein extracts from animal tissues were previously disaggregated and processed in similar fashion.

### mRNA Extraction, RT, and Real-time PCR Analysis

RNA extraction was performed using Tri Reagent (Molecular Research Center). Two micrograms of total RNA were reverse transcribed following the instructions for the high-capacity cDNA RT (Thermo Fisher) protocol, and samples were stored at −70°C (36). The following TaqMan FAM/MGB probes were purchased from Applied Biosystems: human Rcan1 (Hs01120954_m1), human Rcan1.1 (Hs01120956_m1), human PTGS2 (Hs00153133_m1), human MYLK (Hs00364926_m1), human NOS3 (Hs01574659_m1), human ROCK1 (Hs01127699_m1), and human CAMK2B (Hs00365799_m1). Human Rcan1.4 was purchased customized according to primer forward: GCAAACAGTGATATCTTCAG CGAAA, primer reverse: GTGATGTCCTTGTCATACGTCCTAA, and the labeled CAGGGCCAAATTT (5 NFQ). Reactions were incubated in the presence of AmpliTaq Gold DNA polymerase (Applied Biosystems) for 2 min at 50°C followed by 10 min at 95°C. Reactions were then run over 40 cycles of 95°C for 15 s and 60°C for 1 min. Human beta actin VIC/MGB or 18s VIC/MGB rRNA transcripts were used as an internal control, which were amplified in the same tube to normalize for variation in input RNA. The amount of target mRNA in the samples was estimated by the 2CT relative quantification method. Ratios were calculated between the amounts of mRNA from stimulated and/or transfected vs nonstimulated control ECs.

### *In Vitro* Vascular Permeability Assays

Endothelial barrier integrity was evaluated by using Transwell 24-well cell culture inserts (TW) including a membrane pore size of 0.4 µm (Corning). ECs were seeded at a density of 10^5^ cells/well in TWs previously coated with 0.5% gelatin diluted in sterile water and grown in DMEMF12 media supplemented as described above. After several days, EC monolayers were observed and starved for 18 h before the experiments were performed. Stimulus together with a final concentration of 1 mg/ml of FITC-Dextran (Sigma) was added to the upper chamber. Vascular endothelial-permeability measurements were determined by measuring fluorescence of the recipient at 5–30 min and 2 h. All samples were evaluated at least in duplicate.

### Lentiviral Production and Infection

Lentiviruses expressing Rcan1-1-IRES-GFP, Rcan1-4-IRES-GFP, and IRES-GFP were obtained by transient calcium phosphate transfection of HEK-293. The supernatant containing the lentiviral particles was collected 48 h after removal of the calcium phosphate precipitate, filtered through a 45-µM PVDF membrane (Steriflip; Millipore), and ultracentrifuged for 2 h at 26,000 rpm at 4°C (Ultraclear Tubes, SW28 rotor, and Optima l–100 XP Ultracentrifuge; Beckman Coulter). Viruses were resuspended, titrated, and infection efficiency (GFP-expressing cells) was monitored by flow cytometry (36). Lentiviral infection was performed in subconfluent primary cultures of ECs with a 1:1 mix including Rcan1-IRES-GFP (Rcan1-1-IRES-GFP + Rcan1-4-IRES-GFP) or IRES-GFP. Cells were exposed to the lentiviruses in the presence of 10% FBS during 5 h (37). After 4 days, cells were starved of FBS (0.5%) during for 18 h and the experiments of interest were performed.

### Animal Experimental Designs

Animal procedures were carried out in accordance with the European Union guidelines for the care and experimental use of animals. Protocols with reference PROEX: 391/15 received prior approval from the IIS-FJD Ethics Committee and the competent authorities in the region of Madrid.

Two-month-old C57BL/6J mice were intravenously (i.v.) injected with Evans blue (0.04 µg/g in NaCl) followed by subcutaneous (s.c.) injection with histamine and PAF at 5–50 ng/ml for 10 min. To evaluate systemic vascular permeability, histamine at 10 mg/kg or PAF 2 µg/g were i.v. injected together with Evans blue dye. Once i.v. administrated, mice were sacrificed after 15 min, and most PAF animals died spontaneously. The skin pieces, aortas, lungs, and hearts of these animals were incubated in 500 µl of formamide at 55°C for 48 h, and the Evans blue content was determined by absorption at 595 nm. To test passive systemic anaphylaxis (PSA) using an experimental *in vivo* model, mice were i.v. injected with 20 µg of anti-DNP IgE. After 24 h, the mice were challenged with an i.v. injection of 1 mg DNP-HSA (human serum albumin) for the development of anaphylaxis. To test for active systemic anaphylaxis (ASA), we used the classical model, sensitizing mice with i.p injection of 1 mg BSA and 300 ng pertussis toxins as adjuvant in normal saline. After 14 days, the mice were challenged with i.v. injection of 2 mg BSA (38). Following administration of s.c. and i.v. histamine, PSA and ASA mice were sacrificed 30 min after the challenge by cervical dislocation, and blood sampling and biopsies (organ collection) were collected for molecular analysis. All studies were repeated at least once to assure reproducibility. *Rcan1*-deficient (−/−) mice were generated as previously described (39). All mice were genotyped by PCR of tail samples using the following primers: Rcan1, 5′-GGTGGTCCACGTGTGTGAGA-3′, 5′-ACGTGAACAAAG GCTGGTCCT-3′, and 5′-ATTCGCAGCGCATCGCCTTCTAT CGCC-3′. Control littermates were used in all experiments.

### Statistical Analysis

All values are expressed as the mean ± SEM. Differences were evaluated with GraphPad Prism 7.3 program using one-way ANOVA analysis followed by Bonferroni’s *post hoc* test (experiments ≥3 groups) or Student’s *t*-test (experiments with two groups). Statistical significance was set at *p* < 0.05.

## Results

### Histamine Increases Rcan1-4 Expression in HV-EC

**Figure 1:**
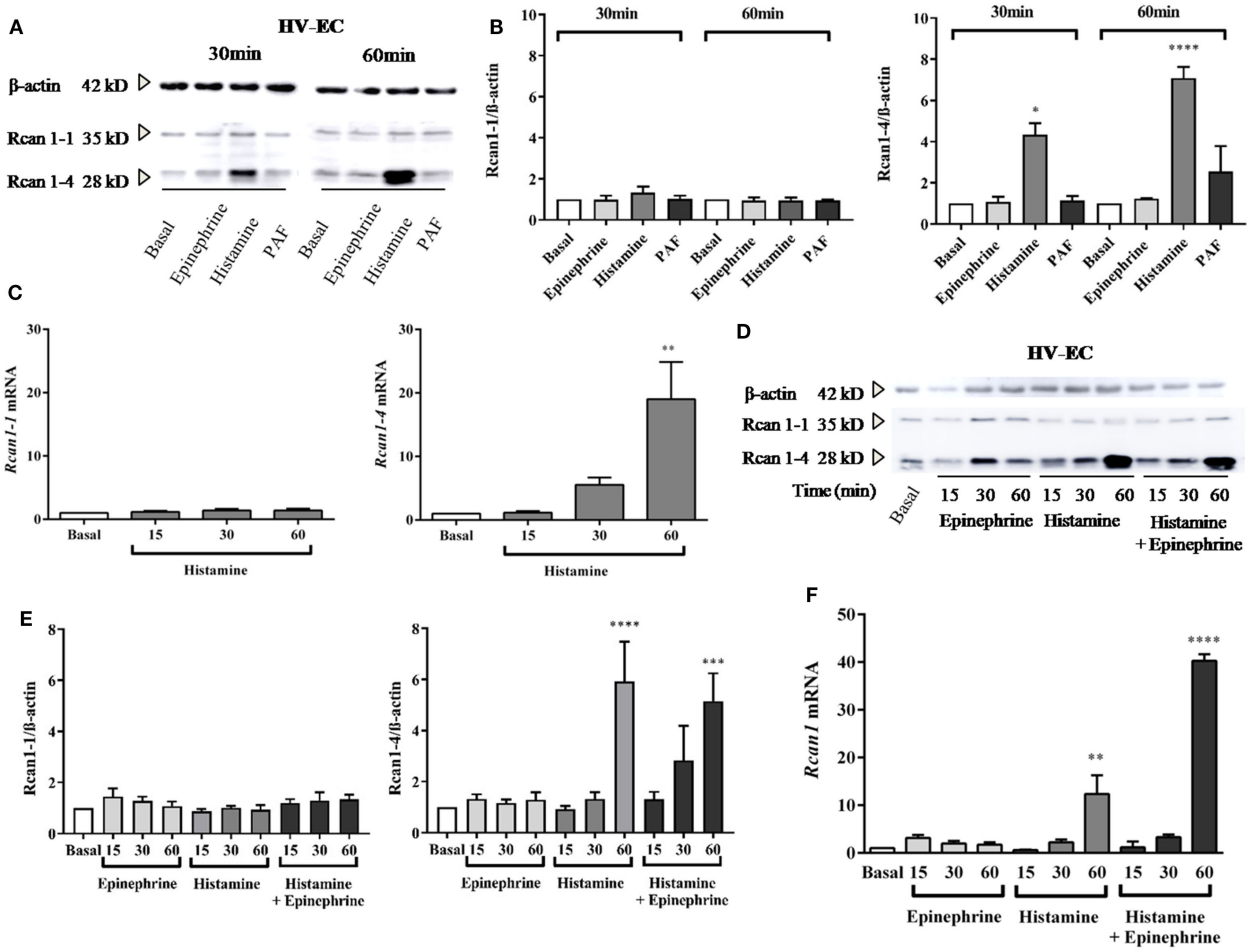
Histamine increases Rcan1-4 protein and mRNA expression in human vein (HV)-endothelial cells (ECs). HV-ECs were treated with epinephrine (1 µM), histamine (1 µM), or platelet-activating factor (PAF) (0.1 µM) at indicated times. **(A)** Representative immunoblots show Rcan1-1 (35 kDa) and Rcan1-4 (28 kDa) expression in stimulated extracts. **(B)** Quantifications were normalized to β-actin and expressed as times of relative change to nonstimulated (basal) cells. Data represent means ± SEM of three and four experiments performed at 30 and 60 min, respectively. One-way ANOVA followed Bonferroni’s multiple comparisons test was performed for each time studied [*P = 0.0375 vs basal (30 min), ****P < 0.0001 (60 min)]. **(C)** qPCR analysis of *Rcan1-1* and *Rcan1-4* mRNA with indicated stimulus and times normalized to the endogenous 18s gene. Data represent means ± SEM of four experiments performed at 15, 30, and 60 min. One-way ANOVA followed Bonferroni’s multiple comparisons test was performed (**P = 0.0052 vs basal). **(D–E)** HV-ECs were treated with epinephrine, histamine, and histamine in the presence of epinephrine at indicated times. **(D)** Representative immunoblots. **(E)** Quantifications were normalized to β-actin and expressed as times of relative change to nonstimulated (basal) cells. Data represent means ± SEM of five experiments performed at 15, 30, and 60 min. One-way ANOVA followed Bonferroni’s multiple comparisons test was performed (****P < 0.0001, ***P = 0.0009 vs basal). **(F)** qPCR analysis of total *Rcan1* mRNA with indicated stimulus and times normalized to endogenous gene expression. Data represent means ± SEM of six experiments performed at 15, 30, and 60 min. One-way ANOVA followed Bonferroni’s multiple comparisons test was performed (****P < 0.0001, **P = 0.0019).

Previous studies have demonstrated the stimulation of Rcan1-4 protein induced by agents that cause the mobilization of Ca^2+^ (33, 34, 36). We observed that histamine stimulation induces a specific increase in Rcan1-4 but not Rcan1-1 protein after a short period (30 min of stimulation); the increase was considerably greater after 60 min of exposure in HV-ECs (Figures 1A,B). Parallel to these results, *Rcan1-4* mRNA expression also increased during the same time frame in response to histamine (Figure 1C). Moreover, as histamine is not the sole mediator recognized for anaphylaxis, we addressed the effect of PAF on HV-ECs. Over the last decade, evidence based primarily on animal models has indicated the existence of an anaphylaxis reaction mediated by IgG- FcγRIII (5, 40), and PAF has been recognized as essential in those non-IgE-mediated reactions (41). In our studies, PAF did not modify Rcan1-4 protein expression in HV-ECs (Figures 1A,B). Additionally, since epinephrine/adrenaline is the first-line treatment in anaphylaxis (1, 42), we set out to determine whether epinephrine could modulate Rcan1-4 expression in ECs. Incubation of epinephrine from 15 min to 1 h did not modify Rcan1-4 protein expression; however, histamine and epinephrine coincubation increased the expression of the Rcan1-4 protein in HV-ECs (Figures 1D,E). As with the protein findings, *Rcan1* mRNA expression increased in response to histamine and epinephrine (Figure 1F). In all cases, the Rcan1-1 protein and mRNA isoform was unchanged in its expression.

### Histamine Increases Rcan1-4 Expression in HA-EC, HMVEC-D, and Lungs of Experimental Anaphylaxis

**Figure 2:**
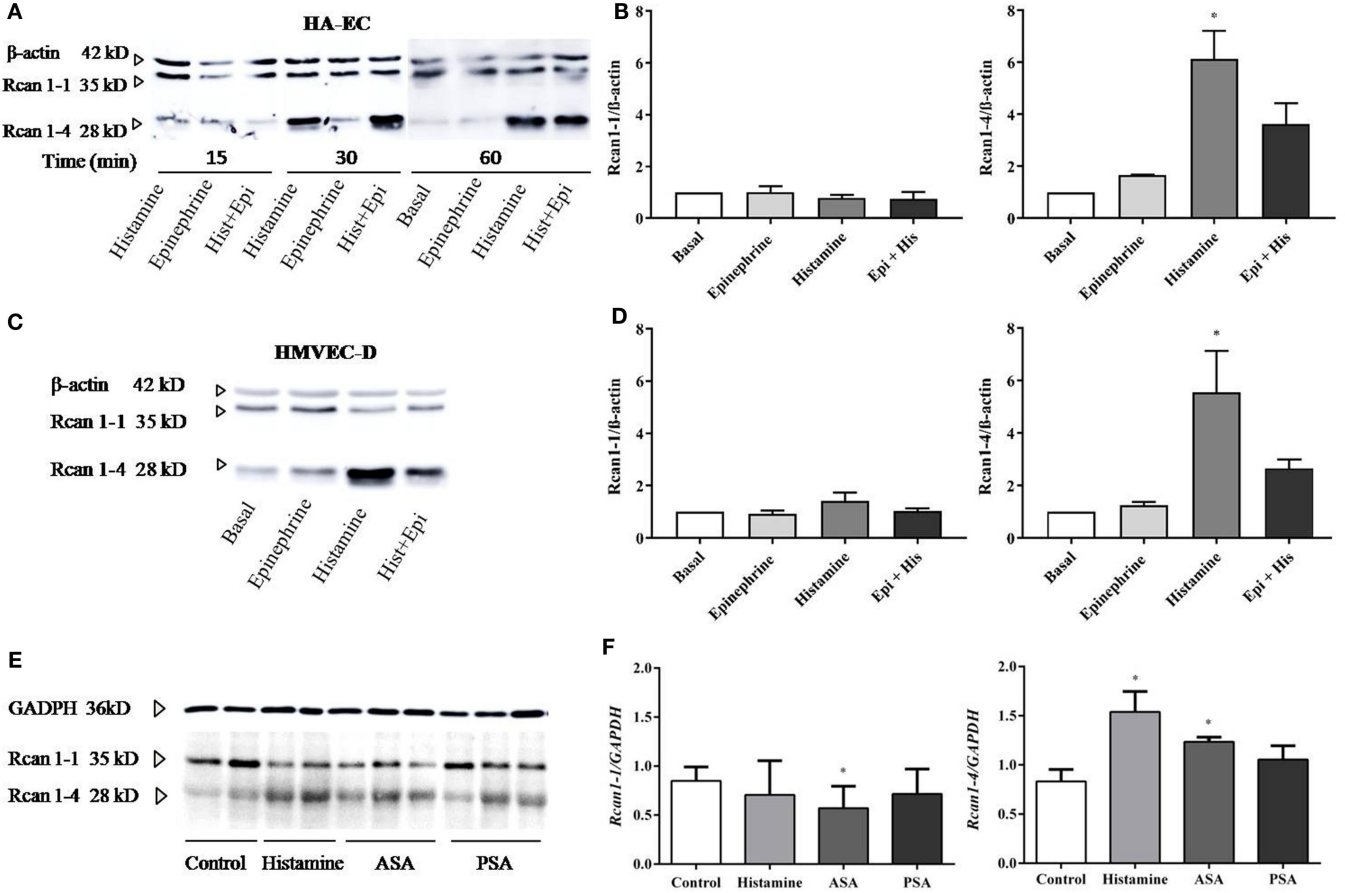
Rcan1-4 expression is modulated in response to histamine in other cellular microenvironments—HA-ECs, HMVEC-D, and lungs from experimental anaphylaxis. HA-ECs and HMVEC-D were treated with epinephrine (1 µM) and/or histamine (1 µM). **(A)** Representative immunoblots show Rcan1-1 (35 kDa) and Rcan1-4 (28 kDa) expression in stimulated HA-ECs for 15, 30, and 60 min. **(B)** Quantifications were normalized to β-actin and expressed as times of relative change to nonstimulated (basal) cells. **(C)** Representative immunoblots show Rcan1-1 (35 kDa) and Rcan1-4 (28 kDa) expression in HMVEC-D for 60 min. **(D)** Quantifications were normalized to β-actin and expressed as times of relative change to nonstimulated (basal) cells. Data represent means ± SEM of five experiments performed per cellular type at 60 min. One-way ANOVA followed Bonferroni’s multiple comparisons test was performed for each cellular type studied (HA-ECs; *P = 0.0115, vs basal, HMVEC-D; *P = 0.0135, vs basal). **(E)** Immunoblots include representative lung extracts from wild-type control mice C57BL6, injected i.v. with Hist, active systemic anaphylaxis (ASA), or passive systemic anaphylaxis (PSA). **(F)** Quantification was normalized to GAPDH and expressed as times of relative change to control mice. Data represent means ± SEM of five animals per group. Unpaired *t*-tests were performed vs control (Rcan1-1 *P = 0.0462; Rcan1-4 *P < 0.02).

The cellular heterogeneity of the whole endothelial compartment has been recognized for some time. Both intracellular mechanisms and functional abilities may be different between the micro-, artery, or vein ECs (43, 44). For this reason, we next checked Rcan1-4 modulation by histamine and epinephrine in other endothelial vascular microenvironments. By using primary artery-derived ECs (HA-EC), our results showed that the contact with histamine induced a marked increase in Rcan1-4 protein expression after 30 min and 1 h (Figures 2A,B), which was correlated with elevated levels of *Rcan1-4* mRNA expression (data not shown). As we previously observed in HV-ECs, epinephrine had no effect on Rcan1-4 expression when incubated alone; however, coincubation with histamine induced a high Rcan1-4 increase in HA-ECs. Additionally, experiments addressing Rcan1 expression in human dermal microvascular ECs (HMVEC-D) showed similarly increased levels of Rcan1-4 protein and mRNA expression in response to histamine. Contrary to this, no effect was observed upon epinephrine stimulation within the studied time frame in HMVEC-D (Figures 2C,D). These results suggest that histamine modulates Rcan1-4 expression in ECs of large vessels regardless of whether they are veins, arteries, or the microvasculature.

The molecular and physiological differences found between studies in animals and studies in humans have sparked debate in anaphylaxis as well as other fields of research (5). Using three experimental designs simulating different degrees of allergic sensitivity, we looked for Rcan1 expression in target organs in mice undergoing anaphylaxis. Experimental passive anaphylaxis (PSA) is based on systemic IgE anti-DNP sensitization over 24 h, while ASA was induced by BSA followed by challenge 14 days later. Analysis of Rcan1 expression in lung extracts of mice treated with histamine for 30 min or undergoing PSA or ASA showed increased Rcan1-4 levels compared to lungs of control mice (Figures 2E,F).

### Rcan1-4 Protein Expression Is Induced by Histamine *via* Its H1 Receptor and a Calcineurin-Dependent Mechanism

**Figure 3:**
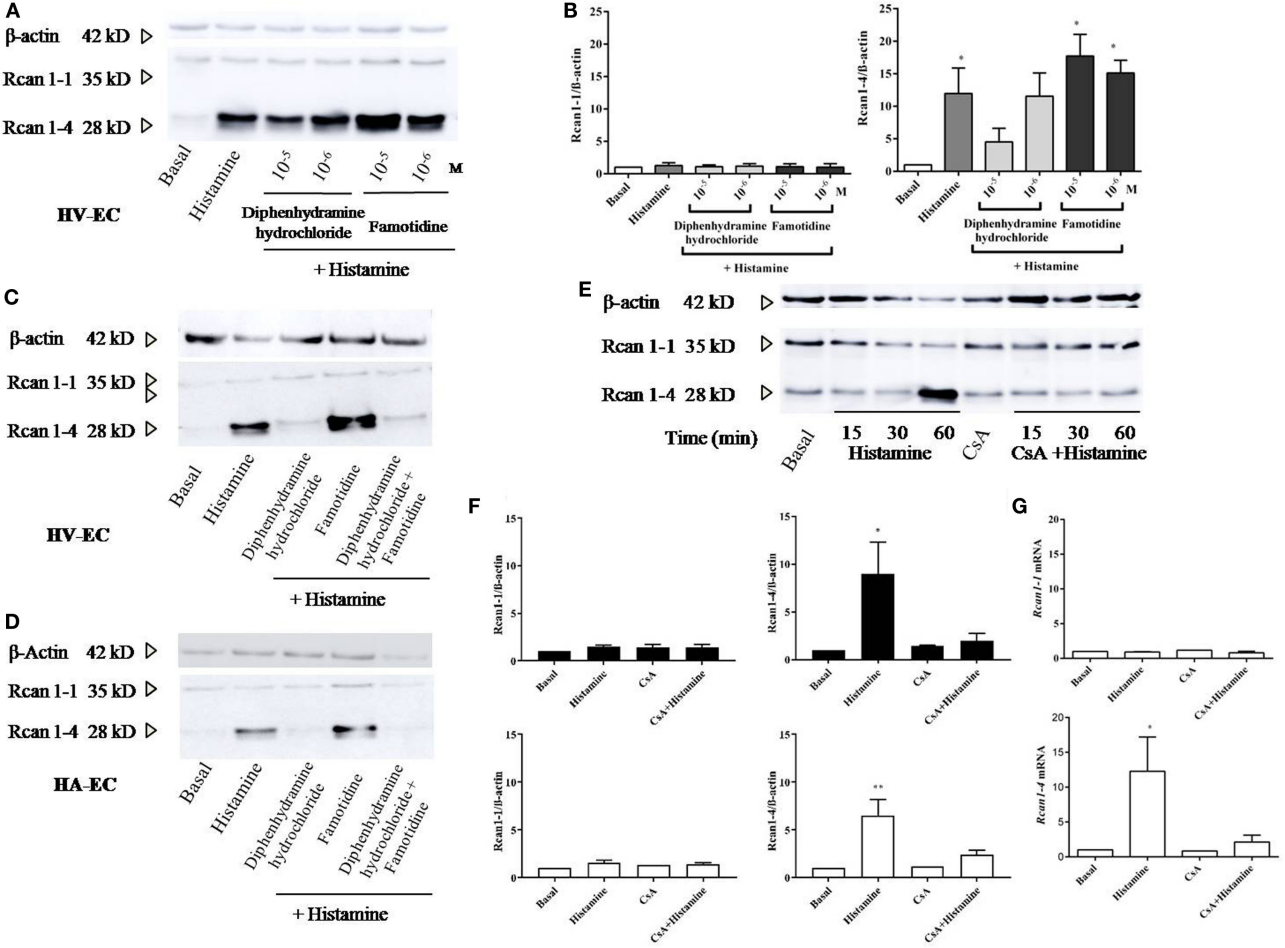
Histamine induces Rcan1-4 protein expression *via* its H1 receptor and CsA sensitive manner. Cells were preincubated with diphenhydramine hydrochloride (H1 receptor antagonist) and famotidine (H2 receptor antagonist) for 30 min at indicated concentrations previously to histamine 1 µM stimulation. **(A)** Representative immunoblots show Rcan1-1 (35 kDa) and Rcan1-4 (28 kDa) expression in stimulated human vein (HV)-endothelial cells (ECs). **(B)** Quantifications were normalized to β-actin and expressed as times of relative change to nonstimulated (basal) cells. Data represent means ± SEM of five experiments performed. One-way ANOVA followed Bonferroni’s multiple comparisons test was performed (*P < 0.05). **(C,D)** Representative immunoblots show Rcan1-1 (35 kDa) and Rcan1-4 (28 kDa) expression in stimulated HV-ECs and HA-ECs preincubated with diphenhydramine hydrochloride, famotidine and a combination of both at a concentration of 10^−5^ M. **(E,F)** Rcan1 immunoblot in extracts from HV-ECs stimulated with histamine 1 µM at indicated times after pretreatment as indicated (30 min) with 200 ng/ml CsA (cyclosporine A). Representative immunoblots show Rcan1-1 (35 kDa) and Rcan1-4 (28 kDa) expression in stimulated HV-ECs. **(F)** Quantifications were normalized to β-actin and expressed as times of relative change to non-stimulated (basal) cells. Data represent means ± SEM of five and six experiments, respectively, performed on HV-ECs (black bars) and HMVEC-D (white bars) at 60 min. One-way ANOVA followed Bonferroni’s multiple comparisons tests were performed (HV-ECs *P = 0.0232; HMVEC-D **P = 0.0022). **(G)** qPCR analysis of *Rcan1-1* and *Rcan1-4* mRNA with indicated stimulus normalized to the endogenous 18s gene. Data represent means ± SEM of four experiments performed at 60 min. One-way ANOVA followed Bonferroni’s multiple comparisons test was performed (*P = 0.0197 vs basal).

Studies were performed to evaluate the relevant receptors (H1R/H2R) involved in histamine-induced Rcan1-4 expression. Prior to stimulation with histamine, HV-ECs were incubated with increasing concentrations of diphenhydramine hydrochloride (type 1 receptor antagonist), determining the optimal concentration of use at 10^−5^ M. Cellular pre-incubation with diphenhydramine hydrochloride completely blocked Rcan1-4 expression induced by histamine in HV-ECs (Figures 3A–C). In contrast, no inhibitory effect mediated by famotidine (type 2 receptor antagonist) was observed; indeed, an additional increase in Rcan1-4 expression induced by histamine stimulation was noted when the receptor type 2 was blocked (Figures 3A–D). Additionally, coincubation with antagonists for both receptors (diphenhydramine hydrochloride plus famotidine) abolished Rcan1-4 expression induced by histamine in HV-EC and HA-ECs (Figures 3C,D).

Next, we investigated the potential role of calcineurin in mediating Rcan1-4 expression induced by histamine. Cellular pre-incubation with CsA completely blocked Rcan1-4 expression induced by histamine in HV-ECs and HMVEC-D (Figures 3E,F). Similar to the protein findings, *Rcan1-4* mRNA expression was significantly diminished in the presence of CsA (Figure 3G). In addition, an increase in calcineurin phosphatase activity is observed in response to histamine in HMVEC-D (data not shown).

### Human Rcan1 Induces Marked Prevention in Vascular Permeability, Strengthening the Endothelial Contact

**Figure 4:**
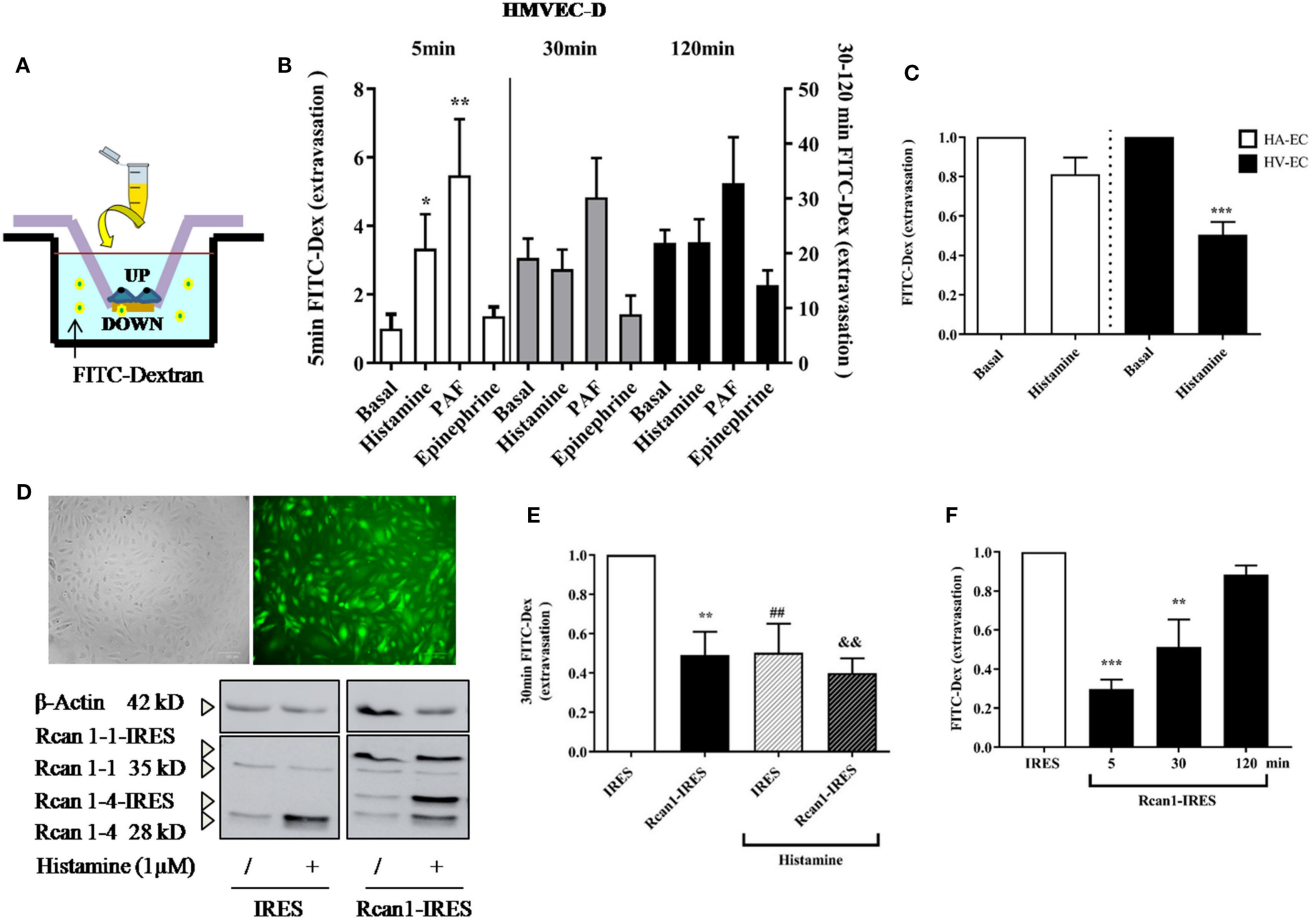
Regulator of calcineurin 1 (Rcan1) maintains the endothelial barrier integrity in human vein (HV)-endothelial cells (ECs). **(A)** Integrity of endothelial monolayers was evaluated using TW assays. **(B)** Quantification of FITC-Dextran molecules extravasated to the TW container and expressed as times of relative change to untreated (basal) cells for 5 (left bars), 30, and 120 min (right bars). Data represent means ± SEM of duplicates determined by TW in five independent experiments performed on HMVEC-D. One-way ANOVA followed Bonferroni’s multiple comparisons tests were performed for each time studied (*P < 0.05, **P < 0.001). **(C)** Quantifications expressed as times of relative change to untreated HV-ECs or HA-ECs, respectively, for 30 min. Data represent means ± SEM of duplicates determined by TW in five independent experiments performed in each genotype. Unpaired *t*-tests were performed vs basal cells (***P = 0.0003 vs basal HV-ECs). **(D)** The correct infection with lentiviral constructions expressing IRES-GFP or Rcan1-GFP were detected by fluorescence microscopy and analyzed by Western blot. The panel shows Rcan1-IRES-GFP extracts modified by infection expressing both exogenously overexpressed isoforms (Rcan1-1 and Rcan1-4). **(E)** Quantification of FITC-dextran molecules extravasated to the TW container and expressed as times of relative change to IRES-GFP cells not treated with histamine 1 µM for 30 min. Data represent means ± SEM of duplicates determined by TW in six (IRES-GFP) and seven (Rcan1-IRES-GFP) independent experiments performed on HV-ECs. One-way ANOVA followed Bonferroni’s multiple comparisons tests was performed (**P < 0.0057, ^##^*P* < 0.0094, ^&&^*P* < 0.0012 vs IRES-GFP control). **(F)** Quantification of FITC-dextran molecules extravasated to the TW container and expressed as times of relative change to IRES-GFP cells for 5, 30, and 120 min. Data represent means ± SEM of duplicates determined by TW in three independent experiments performed on HV-ECs. One-way ANOVA followed Bonferroni’s multiple comparisons tests was performed (***P = 0.0007, **P = 0.0069 vs IRES-GFP).

*In vitro* ECs adhere to coated surfaces, making up the endothelial monolayer. To evaluate vascular permeability *in vitro*, we employed available assay systems (Transwell, TW) based on measurements of extravasations of fluids through an endothelial monolayer. These cells supported on permeable membranes are inserted in individual containers that allow the transport of molecules through them (Figure 4A). First, it was verified that the system allows a measurable increase of FITC-Dextran over time. EC monolayer blocks the passage of the dye in resting conditions (and only endothelial permeability is increased throughout the course of the experiment). As expected, short incubations (minutes) with the vasoactive mediators histamine and PAF induced a rapid increase in vascular permeability on HMVEC-D, which was not observed by incubation with epinephrine (12). The response duration and sensitivity induced by histamine may vary depending on ECs (45). After 15–30 min, we observed a clear tendency to block the passage of molecules across the endothelial barrier both in the presence of histamine and epinephrine, indicating a transient effect of histamine on vascular permeability followed by cell dilation. At this time, only PAF continued destabilizing the endothelial barrier (Figure 4B). Following this, we checked the histamine-induced barrier effects in ECs of big vessels. Our studies did not show modification in barrier properties after short periods of stimulation with histamine in HV-ECs or HA-ECs. However, a clear cell-dilating effect induced by histamine is observed in HV-ECs, and a tendency toward this same dynamic was seen in HA-ECs (Figure 4C). In order to determine whether Rcan1 has a functional contribution in these processes, we used lentiviral constructs aimed at exogenously modifying Rcan1 expression on ECs. HV-ECs were transduced with Rcan1-IRES-GFP or the lentiviral control IRES-GFP. The efficiency of infection in human cell cultures was analyzed by GFP expression using flow cytometry (data not shown), fluorescence microscopy, and Western blotting (Figure 4D). Lentiviral infection does not modify the effects of histamine on cells, as the Rcan1 pattern expression showed the increase of Rcan1-4 protein resembling that seen in non-transduced cells for both IRES-GFP and Rcan1-IRES-GFP HV-ECs (Figures 1–3).

To evaluate the role of Rcan1 in vascular permeability, IRES-GFP and Rcan1-IRES-GFP HV-ECs were seeded in TW and stimulated with or without histamine for 30 min. Data show that Rcan1 overexpression prevents basal FITC extravasation in HV-ECs, indicating a marked role of Rcan1 in blocking cell permeability. In addition, HV-ECs infected with control vector and exposed to histamine produced an effect resembling the one observed previously in non-transduced cells. However, no additional effects of histamine were observed when the cells overexpressed Rcan1 endogenously (Figure 4E). To determine whether Rcan1 can also affect the earliest phases of extravasation and its stability over time, time frames of 5 and 120 min were used for the measurements (Figure 4F). Our results indicate that Rcan1 stabilizes the endothelial barrier during short incubation times, though this effect is weakened when incubation is longer.

**Figure 5:**
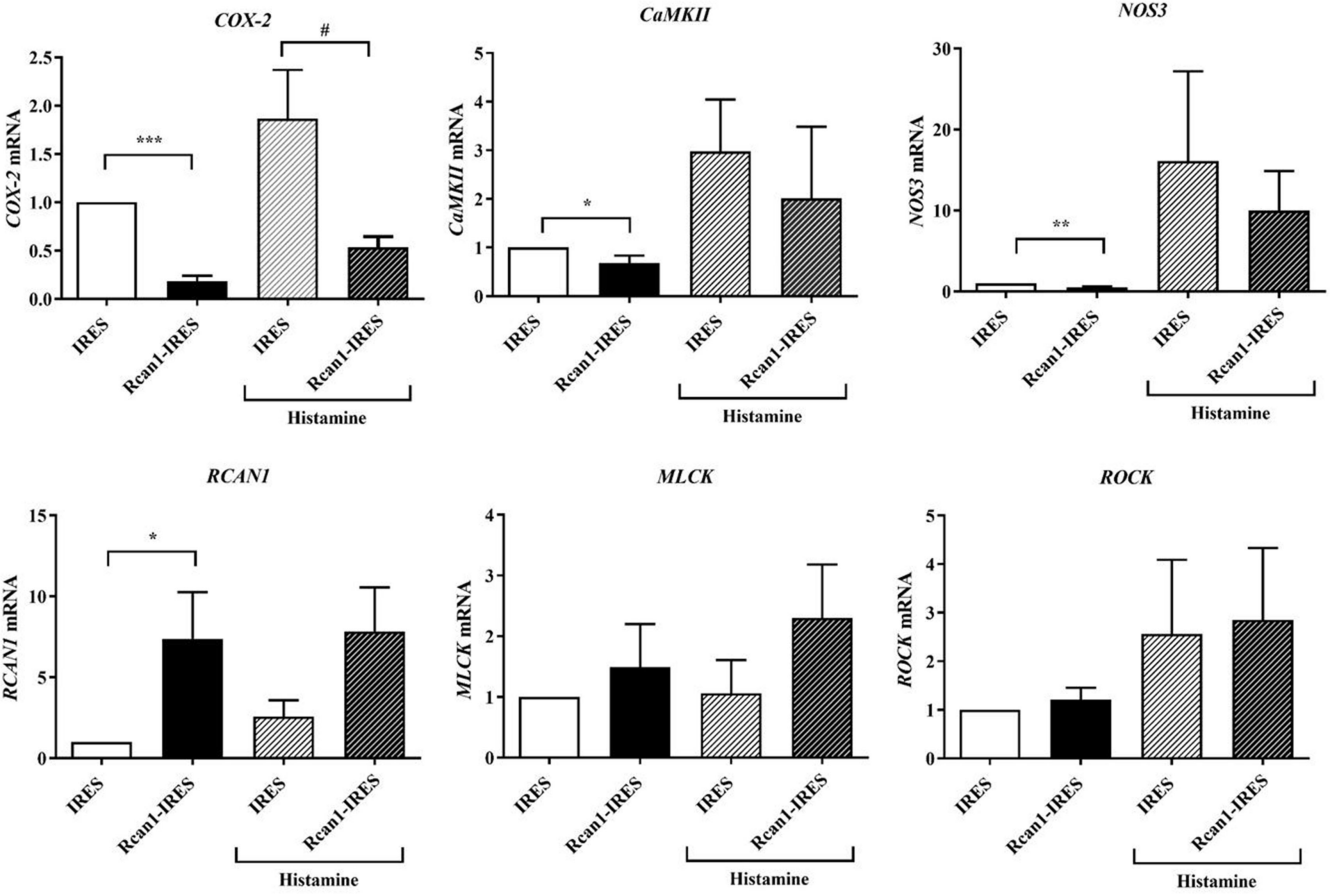
Regulator of calcineurin 1 (Rcan1)-IRES-GFP inhibits *COX2, CaMKII*, and nitric oxide synthase 3 (*NOS3*) expression in human vein (HV)-endothelial cells (ECs). Figure shows the qPCR analysis of *COX2, CaMKII, NOS3*, MLC-kinase (*MLCK*), *ROCK1*, and *RCAN1* mRNA of IRES-GFP and Rcan1-IRES-GFP HV-ECs treated or not with Hist 1 µM for 60 min. Values represent amounts of mRNA normalized to the endogenous gene. Unpaired *t*-test analysis was applied to untreated or histamine-treated cells. Data represent means ± SEM of duplicates determined by qPCR from indicated independent experiments: *COX-2*; ****P < 0.0001, Rcan1-IRES-GFP control (*n* = 13) vs IRES-GFP control (*n* = 12); **^#^***P* = 0.0130, Rcan1-IRES-GFP histamine (*n* = 14) vs IRES-GFP histamine (*n* = 15); *CaMKII*; *P = 0.0454, Rcan1-IRES-GFP control (*n* = 9) vs IRES-GFP control (*n* = 8); Rcan1-IRES-GFP histamine (*n* = 6) vs IRES-GFP histamine (*n* = 6); *NOS3*; **P = 0.0044, Rcan1-IRES-GFP control (*n* = 6) vs IRES-GFP control (*n* = 6); Rcan1-IRES-GFP histamine (*n* = 5) vs IRES-GFP histamine (*n* = 5); RCAN1; *P = 0.0392, Rcan1-IRES-GFP control (*n* = 10) vs IRES-GFP control (*n* = 10); Rcan1-IRES-GFP histamine (*n* = 11) vs IRES-GFP histamine (*n* = 9); *MLCK*; Rcan1-IRES-GFP control (*n* = 9) vs IRES-GFP control (*n* = 10); Rcan1-IRES-GFP histamine (*n* = 8) vs IRES-GFP histamine (*n* = 6); *ROCK1*; Rcan1-IRES-GFP control (*n* = 10) vs IRES-GFP control (*n* = 11); Rcan1-IRES-GFP histamine (*n* = 11) vs IRES-GFP histamine (*n* = 9).

We, therefore, speculated that increased expression of Rcan1 in ECs could in turn modulate other primordial factors involved in anaphylaxis. To test this hypothesis, we analyzed the expression of some genes involved in vascular homeostasis and barrier stability: cyclooxygenase-2 (*COX-2*), calmodulin-dependent protein kinase II-dependent (*CaMKII*), nitric oxide synthase 3 (*NOS3*), MLC-kinase (*MLCK*), and Rho-kinase 1 (*ROCK1*) in resting and histamine-stimulated IRES-GFP and Rcan1-IRES-GFP HV-ECs. Resting Rcan1-IRES-GFP HV-ECs showed a marked inhibition of *COX-2* mRNA, *CaMKII* mRNA, and *NOS3* expression. However, similar levels of *MLCK* and *ROCK1* were observed compared to IRES-GFP HV-ECs (Figure 5). This brief screening suggests that consequent generation of newly formed proinflammatory phospholipid-endothelial derived COX-2 could contribute to vascular contractile processes concomitant to anaphylactic reactions. Additionally, a direct effect on the production of the NO is exerted when Rcan1 is overexpressed.

### Mouse *Rcan1^−/−^* Tissues Present Higher Leakage in Response to s.c. and i.v. Histamine Administration

**Figure 6:**
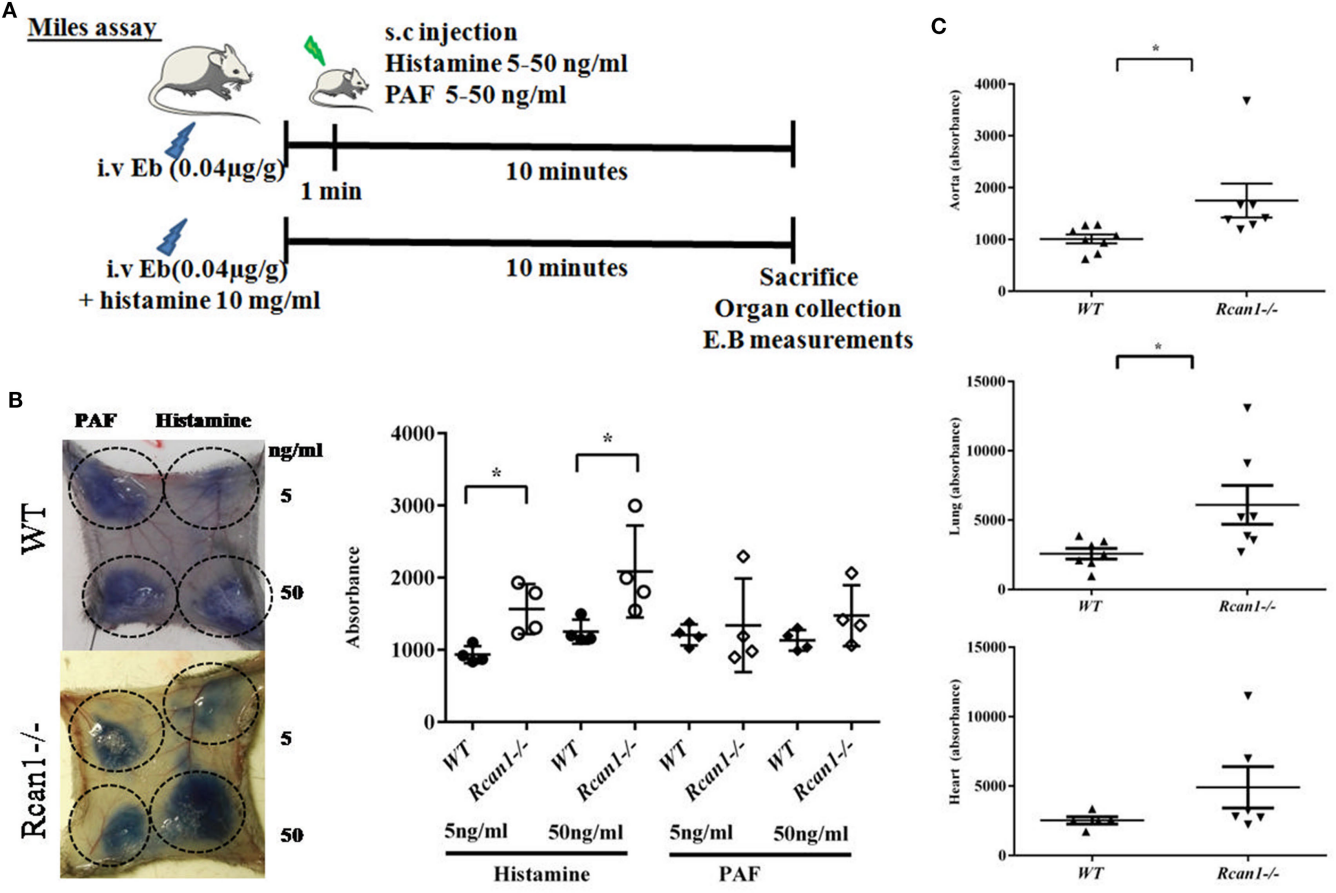
Regulator of calcineurin 1 (Rcan1)-deficient mice prevent histamine-induced vascular subcutaneous and systemic permeability but not systemic anaphylaxis. **(A)** Experimental mice models addressed in *WT* and *Rcan1^−/−^* mice. **(B)** Representative skin pictures of *WT* and *Rcan1^−/−^* mice s.c. injected with mediators at indicated concentrations. Evans blue extravasation was determined in four mice per genotype after subcutaneous injection of 20 µl of the indicated doses of histamine or PAF. Figure shows the amounts of Evans blue determined in skin dorsal punches as described in the Section “Materials and Methods.” Data represent means ± SEM. Unpaired *t*-tests were performed vs *WT* mice for each treatment and doses, *P = 0.0138 vs *WT* (5 ng/ml), *P = 0.0441 vs *WT* (50 ng/ml). **(C)** Graphic shows individual values of aorta and lung extravasation determined in seven (*WT*) and eight (*Rcan1^−/−^)* mice after 15 min of i.v. histamine injection together with Evans blue. Figure shows the amounts of Evans blue determined in tissues as described in the Section “Materials and Methods.” Data represent means ± SEM. Unpaired *t*-tests were performed vs *WT* mice. Aorta; *P = 0.0357, Lung; *P = 0.0319, Heart; ns = 0.1879 compared with Histamine *WT* mice.

The *in vitro* results described above led us to investigate the role of Rcan1 in experimental vascular permeability *in vivo*. We examined the effect exerted by histamine and PAF on vascular permeability in *Rcan1*-deficient vs *WT* mice. NaCl s.c. injection does not modify basal extravasation in *WT* and *Rcan1^−/−^* mice (data not shown). However, an increase of leakage was seen in response to s.c. injection of histamine in both types of mice, and this increase was dose-dependent and significantly higher in *Rcan1^−/−^* mice (Figures 6A,B). Moreover, histamine administrated intravenously increased extravasation in aortas and lungs, though not in the hearts of *Rcan1^−/−^* mice, while no significant difference was observed in response to PAF between genotypes (Figures 6B,C). As in the *in vitro* human cells, endothelial Rcan1 may prevent or recover the loss of endothelial barrier function induced by histamine.

## Discussion

The functional heterogeneity between different EC niches and their importance in the release of certain physiological factors contributing to pathological situations is a topic of high relevance in important fields of research (16, 46). Therefore, in anaphylaxis affecting a large number of organs, it is plausible that the vascular system is a critical participant in the evolution of symptoms and one that may condition the progress of reactions. This study provides the first evidence that histamine, a relevant mediator involved in anaphylactic reactions, modulates Rcan1-4 expression in different human vascular niches (HV-ECs, HA-ECs, and HMVEC-D). Different authors have previously reported that factors such as VEGF and thrombin induce Rcan1-4 expression in cultured ECs (34, 47). Given the major role of histamine when released by immune cells as a mediator of anaphylactic reactions, our results extend previous findings, showing that histamine upregulates Rcan1-4 expression in several types of ECs, whereas Rcan1-1 is not modulated. In agreement with our findings, a number of studies performed on this cellular type did not observe significant Rcan1-1 variation in response to stimuli (34) in spite of its involvement in Huntington disease and mitochondrial autophagy (29, 48). Additionally, epinephrine has no effect on Rcan1 expression, and although its potent actions as a dilator or constrictor through its adrenergic receptors have been widely observed, its role in the endothelium has been poorly investigated. However, the contributions of epinephrine to the maintenance of the endothelial barrier, through its β-adrenergic receptors, have been evaluated in ECs (12). Similarly, a substantial number of studies have demonstrated the role of PAF in anaphylaxis. The data shown here do not support a role for PAF in modulating Rcan1-4 expression in ECs, at least in our experimental conditions. However, given the relevance of PAF, more thorough studies focused on human vascular cells would be of interest to clarify unexplored aspects that thus far have been mostly related to reactions mediated by IgE or IgG antibodies in anaphylaxis (5, 41).

The human H1R acts mainly by coupling to Gq/11 proteins; in fact, experimental anaphylaxis is prevented in endothelial Gq/11-deficient mice (38). Through its specific signal, histamine is one of the most potent vasoactive substances, inducing relaxation or contraction (tissue- and species-dependent) and participating in anaphylactic responses, mainly through its H1R and H2R receptors (8, 9). The data shown here demonstrate that histamine increases Rcan1 expression through the H1R receptor in ECs due to a calcineurin-dependent mechanism, as the calcineurin inhibitor CsA inhibits its expression.

Anaphylaxis is widely recognized by the presence of increased vascular permeability, mediating a shift of intravascular fluid into the extravascular space within minutes, which results in hypotension and hemoconcentration (3). Some studies in human subjects have revealed transient impairment of the microvasculature during severe acute anaphylaxis (49). Thus, the leakage occurring in anaphylaxis requires cellular retraction of ECs as a result of increased cell contractile pathways in response to external stimuli or agents (15). Subsequently, a biological counterbalance between contractile and adhesive forces must exist to maintain the stability between cells or allow cells to recover from the rupture of the endothelial sheet. Thus, most of the mediators described in anaphylaxis elicit vascular EC permeability signaling through specific receptors and molecular pathways. However, some stabilizing molecules preserve the rupture of the endothelial barrier (as cAMP or sphingosine 1 phosphate). The functional evaluation of Rcan1 by using *in vitro* permeability assays and *in vivo* extravasation mice models show Rcan1 as a stabilizer of the endothelial barrier or as a cellular dilator agent. Our studies demonstrate that histamine-induced Rcan1 contributes to the stability of the endothelial barrier and also indicate that this mechanism is a late-secondary response to prevent the loss of fluids and/or the regulation of vascular tone.

Our studies demonstrate that exogenous overexpression of Rcan1 downregulates the expression of calcineurin-related genes such as *COX2, NOS3*, and *CaMKII*. H1R stimulation increases NOS3 synthesis through a mechanism that involves CaMKII in human vascular ECs (24, 50). NOS3 upregulation and consequent NO production is protective under normal conditions, though may be deleterious in a model of experimental anaphylaxis (51, 52). Our studies correlate the stability of the endothelial barrier with a decreased *NOS3* expression in Rcan1 overexpressed cells, as well as a tendency to prevent the increase of *NOS3* induced by histamine, suggesting a protective vasodilator role for Rcan1 in ECs. Interestingly, CaMKII has been reported as a suppressor of intracellular cAMP accumulation (53). The inhibited CaMKII may induce PKA-mediated responses, increasing the levels of cAMP, which in turn could contribute to barrier stability. In fact, direct evidence has demonstrated the ability of Rcan1 to increase the phosphorylation of cAMP response element-binding protein through the negative regulation of the calcineurin signaling pathway (54). Accordingly, Rcan1 overexpression decreases CaMKII levels in our EC system, supporting its role in the strengthening of the endothelial barrier.

Molecular pathways based on disruption of cell–cell contacts and which are regulated by phosphorylation and de-phosphorylation of the myosin light chain are also relevant to this field of research (17, 55, 56). Although RhoA and ROCK have been recently recognized as key targets mediating histamine-induced vascular leakage and anaphylactic shock (14), our results do not reveal significant differences in *MLCK/ROCK1* when Rcan1 is overexpressed in HV-ECs. Nevertheless, the identification of *COX2* as a downstream target of the CN/Rcan1 molecular pathway is a meaningful observation, and one which supports previous studies (57). COX-2 expression is induced by inflammatory stimuli and other mediators in the vascular wall, such as histamine, generating prostaglandin I2, and E2 production in HA-ECs (58). Proinflammatory phospholipid-derived mediators are critical modulators of vascular tone in physiological and pathological situations. COX-2 production induced by histamine has been reported in mast cells (19, 20). In addition to its products, prostaglandin D2, leukotrienes, thromboxane A2, and PAF are released rapidly in anaphylactic events (59, 60). In our studies, *COX2* mRNA was substantially decreased upon histamine stimulation in Rcan1 overexpressed HV-ECs, suggesting a major relevance of the axis histamine/Rcan1-4/*COX2* in anaphylaxis. In general, it can be speculated that both cell types (endothelial and mast cells) may contribute to prostanoid/eicosanoid generation, which greatly render the vasoconstrictor/vasodilator effects that occur in anaphylaxis. Our findings suggest that, through endothelial Rcan1 expression, histamine could also contribute to the regulation of important molecular pathways related to anaphylaxis, although further investigations need to be performed to confirm this.

Knowledge of the consequences of human Rcan1 overexpression is limited to evidence of genetic dysfunctions secondary to trisomy of chromosome 21 in patients with Down syndrome, who present defects of the immune system, though the prevalence of allergy is not high in these individuals (61). Previous studies using experimental anaphylaxis in Rcan1-deficient mice have found a role for Rcan1 in regulating Fc-εRI-mediated signaling and mast-cell function (62). Rcan1^−/−^ bone-marrow-derived mast cells show increased transcriptional activation of NF-κB and NFAT and calcineurin activity following stem-cell factor stimulation (63). On the other hand, Rcan1 seems to be required for the development of pulmonary eosinophilia in allergic inflammation in mice (64). Additionally, Rcan1 plays a protective role for respiratory infections and sepsis in experimental mice models (33, 65). Consistent with this, studies performed on Rcan1 transgenic mouse have found that Rcan1 gives rise to cancer protection by inhibiting the calcineurin pathway in the vascular endothelium (66). Moreover, as an endogenous inhibitor of calcineurin, Rcan1 has been involved as a negative regulator in inflammatory molecular pathways belonging to ECs (47). Our results in human ECs and an experimental mice model support the immunosuppressive function of Rcan1 for vascular permeability and anaphylaxis.

We, here, show that endothelial Rcan1 maintains the integrity of the endothelial barrier in HV-ECs. This effect is closely correlated with the cellular dilatory process that must occur in the resident cells once the primordial effect of endothelial barrier breakdown has been induced by histamine. Extravasation or vascular permeability in micro-ECs may be best understood in terms of cellular contraction and dilatation, though cellular processes do not always correlate with physiology in vessels, and it is plausible that a similar process would occur in the context of anaphylaxis. Our results suggest that Rcan1 overexpression produces cell dilation, at least in HV-ECs, which correlates with the stabilization of the HMVEC-D barrier.

In summary, this work contributes to the knowledge of anaphylaxis, showing endothelial Rcan1 as an inducible molecule which is modulated in the endothelial compartment upon contact with histamine through a calcineurin-sensitive pathway. Additionally, human functional assays and *in vivo* experimental mice models suggest a role for endothelial Rcan1 controlling vascular permeability, most likely to recover the loss of fluids, and pointing to Rcan1 as a plausible regulator of sensitivity to anaphylaxis in humans from the endothelial compartment.

## Ethics Statement

The study was approved by the research ethics committees of the respective Gentofte and FJD hospitals, and written informed consent was obtained from all patients. Animal procedures were carried out in accordance with the European Union guidelines for the care and experimental use of animals. Protocols with reference PROEX: 391/15 received prior approval from the IIS-FJD Ethics Committee and the competent authorities in the region of Madrid.

## Author Contributions

CB-M, NM-B, and VE contributed to the performance of the experimental work; AM-Y and CP-V contributed to the performance of the experimental work included in the reviewed version. BMJ, LHG, LKP and VE as leaders of the overall project “molecular mechanisms in anaphylaxis”; LK, JC-H, and AG-C provided clinical support; NM-B and VE assisted in results interpretation; VE coordinated the work, designed the experiments, and wrote and edited the manuscript; NM-B, BMJ, MG-A, GA-L, CP-V, FV, JC-H, LH-G, and LKP reviewed the manuscript.

## Conflict of Interest Statement

Declarations of transparency and scientific rigor. This Declaration acknowledges that this paper adheres to the principles for transparent reporting and scientific rigor in preclinical research recommended by funding agencies, publishers, and other organizations engaged with supporting research. The authors declare no competing financial interests.
