# Supplementary material for: Endothelial Regulator of Calcineurin 1 Promotes Barrier Integrity and Modulates Histamine-Induced Barrier Dysfunction in Anaphylaxis
Source: Front Immunol. 2017 Oct 20;8:1323. doi: 10.3389/fimmu.2017.01323 (PMC5655011; doi:10.3389/fimmu.2017.01323)
Supplement: Supplementary file 1 [file data_sheet_1.pdf]

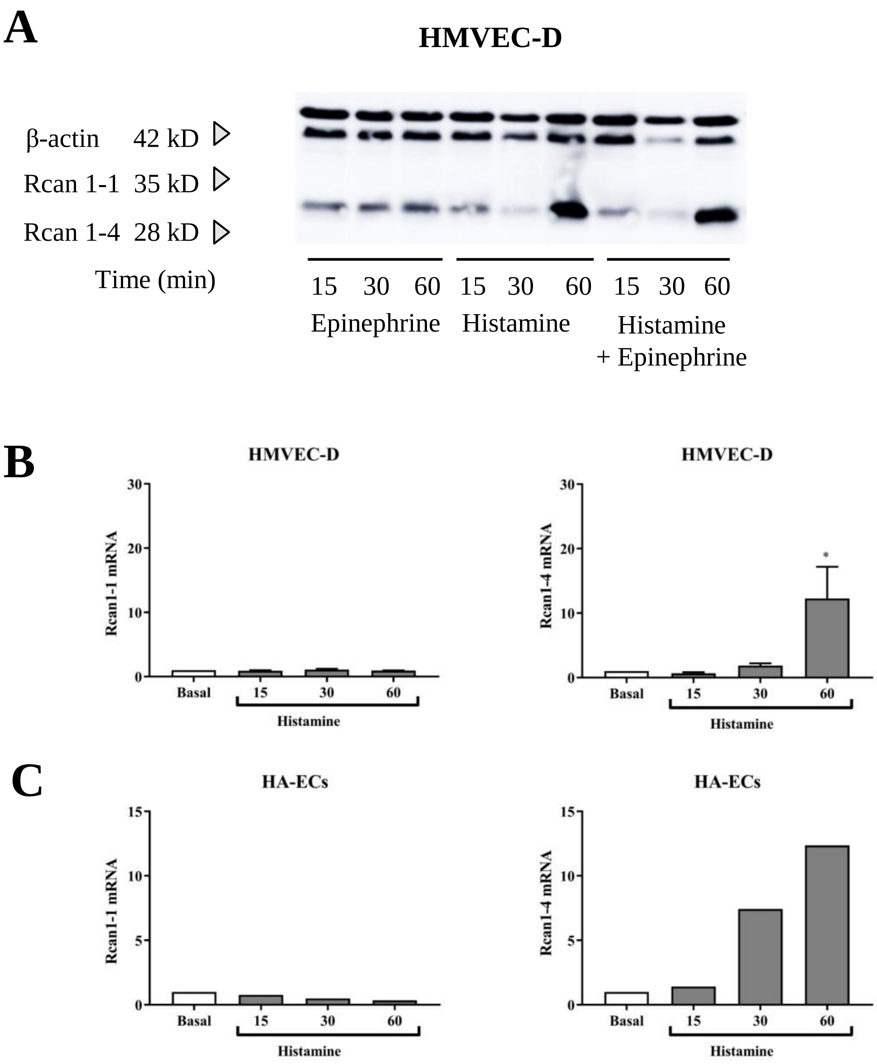

SUPPLEMENTAL FIGURE 1

(A) Incubation of epinephrine from 15 min to 1 h did not modify Rcan1-4 protein expression; however, histamine and epinephrine coincubation increased the expression of the Rcan1-4 protein in HMVEC-D, similarly to HV-ECs (Fig 1D-E) and HAECs (Fig 2A). Figure shows a representative immunoblot.

(B) qPCR analysis of Rcan1-1 and Rcan1-4 mRNA with indicated stimulus and times normalized to the endogenous 18s gene. Data represent means ± SEM of 4 experiments performed at 15, 30 min and 60min in HMVEC-D. One-way ANOVA followed Bonferroni's multiple comparisons test was performed (*P = 0.0470 vs basal).

(C) qPCR analysis of Rcan1-1 and Rcan1-4 mRNA with indicated stimulus and times normalized to the endogenous 18s gene. Data represent 1experiment performed at 15, 30 min and 60min in HAECs.

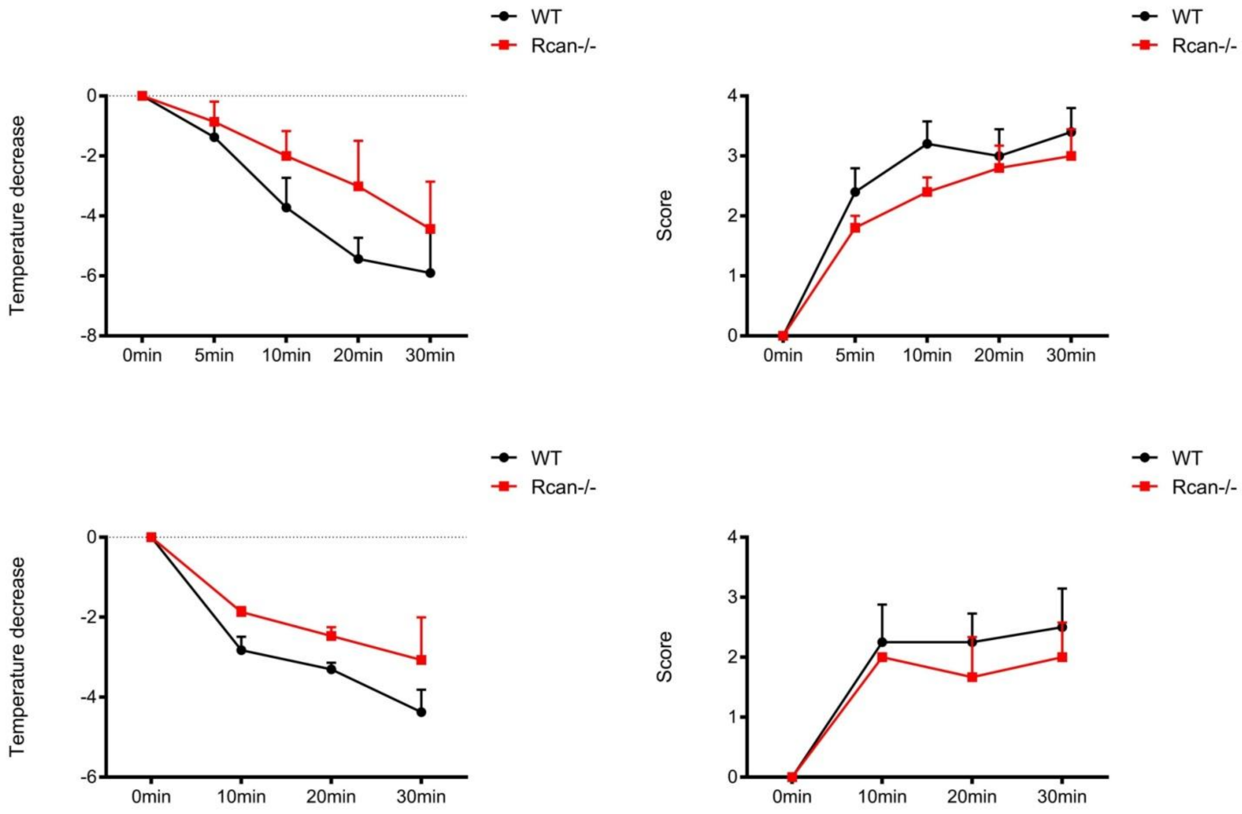

SUPPLEMENTAL FIGURE 2

Active and passive systemic anaphylaxis was performed in 5 WT/5 RCAN1-/- and 4 WT/3 RCAN-/- mice respectively. Rectal temperatures (with a Traceable Digital Thermometer (Model VWR I620-2000) were registered until 30 minutes after challenged. Systemic anaphylactic symptoms in mice were scoring according: 0: no symptoms; 1: scratching and rubbing around the nose and head; 2: puffiness around the eyes and mouth, diarrhoea, ‘pilar erecti’, reduced activity and/ or decreased activity with increased respiratory rate; 3: wheezing, laboured respiration, and cyanosis around the mouth and the tail; 4: no activity after prodding or tremor and convulsion and 5: death

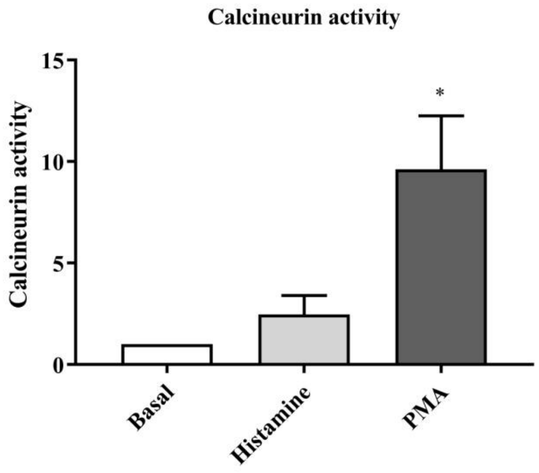

SUPPLEMENTAL FIGURE 3

Figure represents the calcineurin activity analysis with indicated stimulus. Data represent means ± SEM of 3 experiments performed at 1h in HMVEC-D. One-way ANOVA followed Bonferroni's multiple comparisons test was performed (* P =0.0069 vs Basal). Unpaired t-tests was performed for histamine vs basal; P = 0.1899.

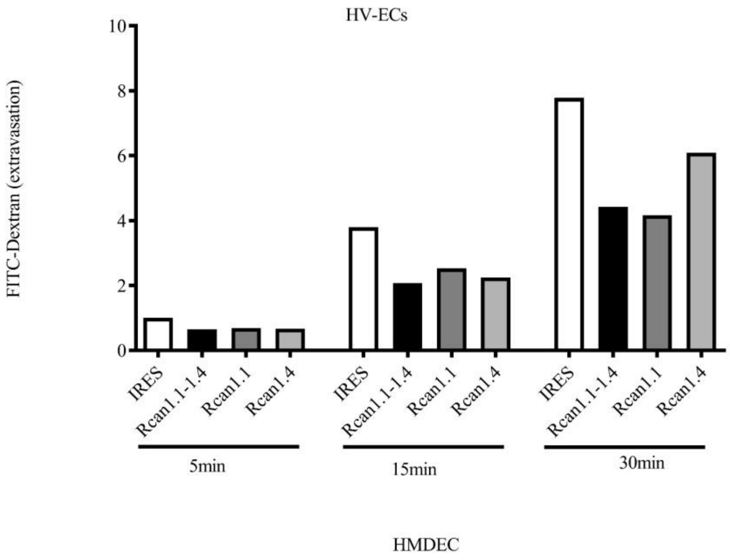

SUPPLEMENTAL FIGURE 4

IRES-GFP, Rcan1L-IRES-GFP+Rcan1S-IRES-GFP, Rcan1L-IRES-GFP and Rcan1S-IRES-GFP constructs have been transfected into HVECs. Figure shows the quantification of FITC-dextran molecules extravasated to the TW container and expressed as times of relative change to IRES-GFP at different times. Data represent means of duplicates determined by TW in one experiment performed.
